# Opto-Mechanical Coupling in Interfaces under Static and Propagative Conditions and Its Biological Implications

**DOI:** 10.1371/journal.pone.0067524

**Published:** 2013-07-04

**Authors:** Shamit Shrivastava, Matthias F. Schneider

**Affiliations:** Department of Mechanical and Biomedical Engineering, Boston University, Boston, Massachusetts, United States of America; UC Davis School of Medicine, United States of America

## Abstract

Fluorescent dyes are vital for studying static and dynamic patterns and pattern formation in cell biology. Emission properties of the dyes incorporated in a biological interface are known to be sensitive to their local environment. We report that the fluorescence intensity of dye molecules embedded in lipid interfaces is indeed a thermodynamic observable of the system. Opto-mechanical coupling of lipid-dye system was measured as a function of the thermodynamic state of the interface. The corresponding state diagrams quantify the thermodynamic coupling between intensity *I* and lateral pressure *π*. We further demonstrate that the coupling is conserved upon varying the temperature *T*. Notably, the observed opto-mechanical coupling is *not* limited to equilibrium conditions, but also holds for propagating pressure pulses. The non-equilibrium data show, that fluorescence is especially sensitive to dynamic changes in state such as the LE-LC phase transition. We conclude that variations in the thermodynamic state (here *π* and *T,* in general *pH, membrane potential V*, etc also) of lipid membranes are capable of controlling fluorescence intensity. Therefore, interfacial thermodynamic state diagrams of *I* should be obtained for a proper interpretation of intensity data.

## Introduction

Lipid membranes are integral parts of biological systems and are consistently under the influence of numerous perturbations. Molecules (e.g proteins) incorporated in the membrane are therefore not only exposed to equilibrium thermodynamic fluctuations, but also to induced non-equilibrium perturbations. We have previously proposed that these perturbations, in the form of propagating density or voltage pulses, may play a major role in inter or intra cellular communication[1]–[3] as well as nerve pulse propagation [4], [5]. Measurements in lipid vesicles have shown that enzymatic activity is a function of the membrane state [6]–[11]. Therefore proteins or enzymes located in the acoustic path of propagating pulses are required thermodynamically to exhibit an altered kinetic behavior. Propagation and excitation of such density waves are difficult to measure due to limitations in detection technologies. One way of probing changes in membrane’s physical properties is by characterizing the emission of fluorescently labeled lipids incorporated in the membrane. Fluorescence emission properties have been extensively studied for different dyes under varying physical conditions. In membranes they have been shown to report chemical and mechanical changes in their surroundings.[12]–[18] Such observations are usually specific to the dye used, its partition coefficient and electronic structure. [17], [19]–[22]. In this work, we study the potential of fluorescently labeled lipids to report non-equilibrium and equilibrium variations in thermodynamic state of the lipid-water-interface in general. Therefore, we thoroughly characterize and quantify the equilibrium and non-equilibrium opto-mechanical coupling of the interface. Our results outline how the intensity of fluorescent emission may be exploited to study propagating density waves in biological membranes.

## Materials and Methods

**Figure 1:**
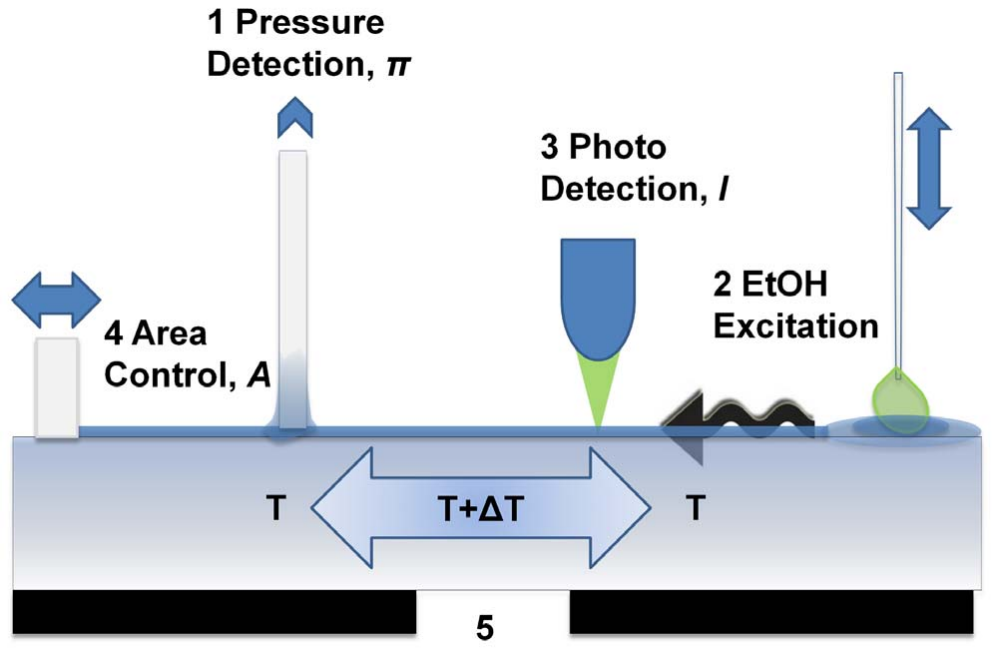
Experimental setup. (1) The lateral pressure is measured using a Wilhemly balance. (2) The monolayer is excited chemically using a dipper that can be moved vertically. (3) Fluorescence is measured simultaneously with pressure. The spot for fluorescence measurement (D = 400 µm) can be moved across the interface to measure fluorescence from different regions (For simultaneous optical and mechanical measurement of a pressure pulse, (1) and (3) were equidistant from (2) at 12cm). (4) The barriers can be moved horizontally to compress the monolayer at the air/water interface. The temperature in the bulk can be controlled from underneath by circulating water from a heat bath (solid black). (5) There is a glass window which introduces local temperature gradients due to the absence of circulating coolant underneath. This gradient was used to investigate the local effects of temperature on the monolayer. At a room temperature of 21°C, on cooling down, the temperature at ‘5′ was 8.5°C compared to 7.7°C in the rest of the trough. The contact of solvent with the monolayer created a trigger signal that was used to control the microscope LED and data acquisition.

Lipids dimyristoyl-sn-glycero-3-phosphocholine (DMPC) and 1,2-dipalmitoyl-sn-glycero-3-phosphocholine (DPPC) dissolved in chloroform were purchased from Avanti Polar Lipids (Birmingham, AL) and used without further purification. Four different lipid conjugated dyes were used in this study. *N*-(7-Nitrobenz-2-Oxa-1,3-Diazol-4-yl) - 1,2 - Dihexadecanoyl - *sn* - Glycero -3-Phosphoethanolamine, Triethylammonium Salt (16∶0 NBD-PE), 1,2-Dihexadecanoyl-*sn*-Glycero-3-Phosphoethanolamine, Triethylammonium Salt (Texas Red DHPE) and *N*-(4, 4 - Difluoro - 5, 7 - Dimethyl - 4 - Bora - 3a, 4a - Diaza- *s* - Indacene - 3 - Propionyl) -1,2-Dihexadecanoyl -*sn*-Glycero-3-Phosphoethanolamine, Triethylammonium Salt (BODIPY FL DHPE) were acquired from Invitrogen. 1,2-dimyristoyl-*sn*-glycero-3-phosphoethanolamine-N-(7-nitro-2-1,3-benzoxadiazol-4-yl) (ammonium salt) (14∶0 NBD-PE) was acquired from Avanti polar lipids. The dyes were diluted with chloroform (>99%, Sigma Aldrich) to obtain final concentration of 0.1% by moles in the lipid solutions. The lipid-dye solutions were spread at the air-water interface in a Langmuir trough (NIMA, UK) to form a monolayer (fig. 1). The temperature of the de-ionized water (18 Mohms) in the sub-phase was kept constant by using a heat bath at a desired temperature while the room temperature was 20°C. The quasi-static equilibrium experiments were performed by compressing the monolayer at two ends with the rate of 28 mm^2^/s with an initial area of 100 cm^2^. A blue LED was used for fluorescence excitation at 465 nm over a spot diameter of 400 µm. The emission intensity was simultaneously measured at 535 nm and 605 nm (BW 10 nm). For the non-equilibrium experiments a bigger trough with a total area of 500cm^2^ was used. The monolayer in this case consisted of 1% NBD in DPPC. The de-ionized water in sub phase was mixed with pure sodium chloride (1 g/l) (Sigma, >99.5%) and was also added to ethanol to get the ethanol:water mixture (2∶1 v/v). Waves were excited by injecting 2 µl of the ethanol-water mixture at the surface using an automated dipper arm. The contact of ethanol droplet with the air-water interface was used to trigger the LED and the data acquisition (10kS/s). The LED was triggered to avoid bleaching and also any signal due to recovery of photo bleaching, as the pulses were accompanied by certain flow in the monolayer. A fresh preparation was used for every excitation event. The optical and mechanical measurements were performed equidistant (12 cm) from the point of excitation.

## Results and Discussion

### 1. Static Opto-mechanical Coupling

**Figure 2:**
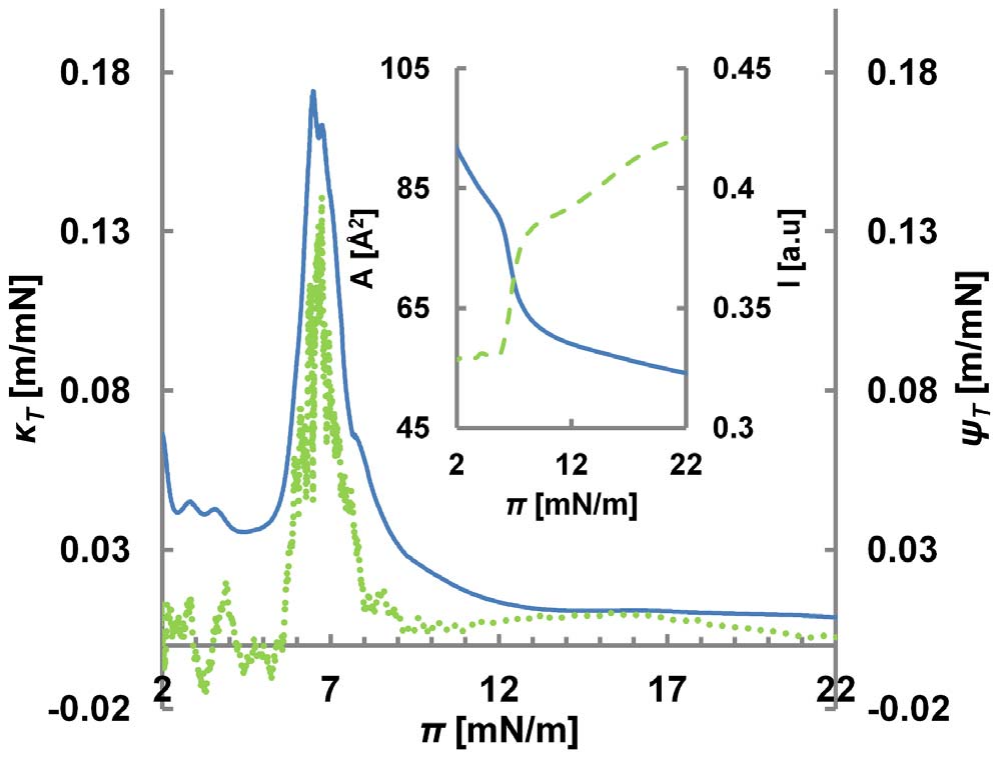
Opto-Mechanical Isotherm for a Quasi-Static Process. Opto-mechanical susceptibility (green-dash) and compressibility (blue – solid), plotted as a function of pressure. The original intensity and area curves have been plotted as a function of lateral pressure in the inset. Area and intensity change nonlinearly as a function of pressure during the LE-LC transition and the peaks in the corresponding susceptibilities (y axis) correlate precisely. The peak in opto-mechanical susceptibility results in increased sensitivity of intensity to pressure variations around LE-LC transition.

In the inset of Fig. 2 both intensity *I* and lateral pressure *π*, as acquired using the set-up shown in Fig. 1, are plotted as a function of lateral pressure *π*. The coupling between the two curves becomes exceptionally pronounced, when plotting the derivatives (Fig. 2), i.e. the compressibility
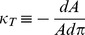
(1)and the opto-mechanical susceptibility
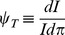
(2)


**Figure 3:**
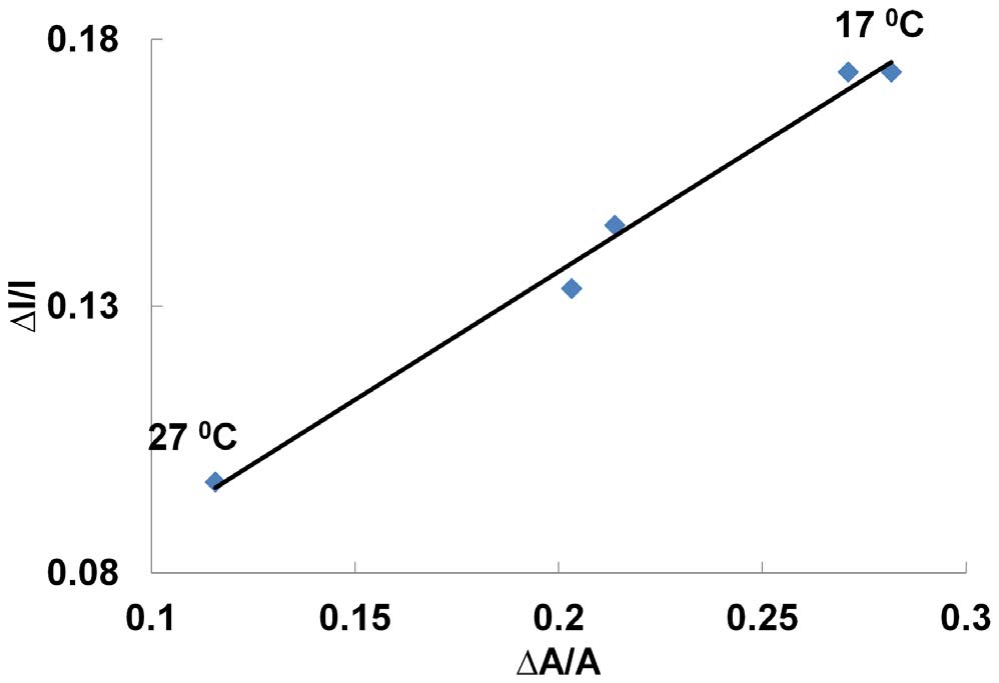
Opto-mechanical Invariance. *(*Δ*I/I)_Tr_* plotted against *(*Δ*A/A)_Tr_* for LE-LC transitions at different temperatures for a DPPC/NBD-PE system. The proportionality between intensity and area is invariant of the temperature. The numerator of the fraction is the total change in the variable during LE-LC transition where as the denominator represents the mean value of the variable during the transition. This also means that intensity like surface area or density is an observable of the interface.

**Figure 4:**
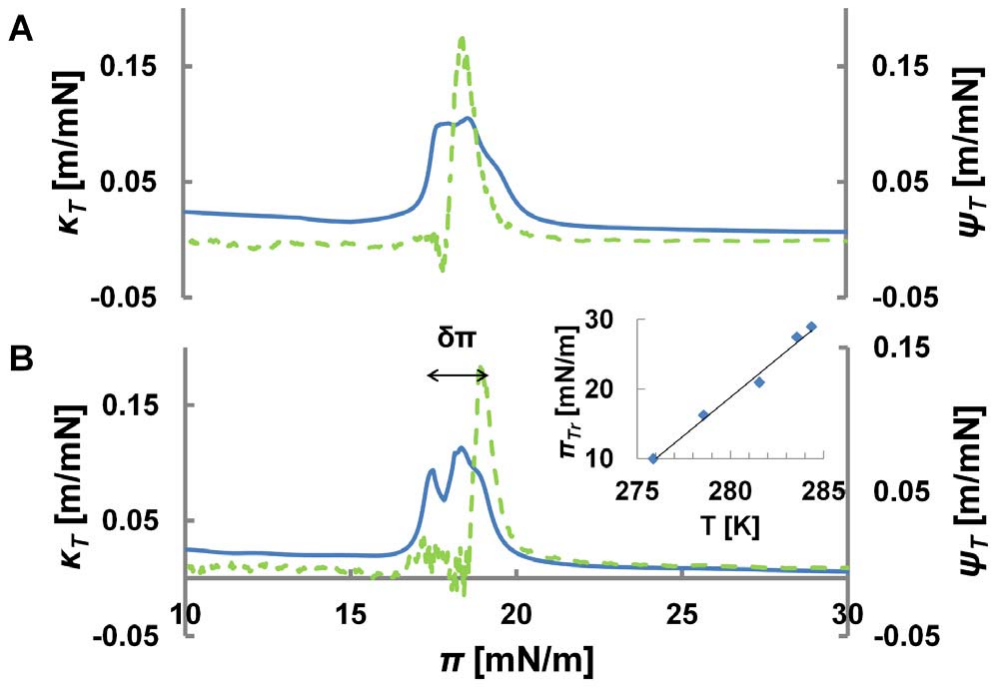
Intensity reports local variations in the mechanical properties of the interface. A) Opto-mechanical susceptibilities of a DMPC-NBD monolayer. The local temperature at the site for optical detection is the same as the average temperature of the monolayer. Consequently the peaks in the corresponding susceptibilities correlate. B) When measured in a region of higher temperature (region 5 in Fig. 1) the opto-mechanic susceptibility (green-dash curve) is shifted against the compressibility (blue-solid curve) by 1.5 mN/m. From the given temperature dependence a local temperature increase by 0.8C (7.78.5C) is expected, which is in perfect agreement with the temperature difference measured during calibration (Fig. 1). The figure in the inset plots a linear regression on transition pressure for isotherms at different temperatures.

**Figure 5:**
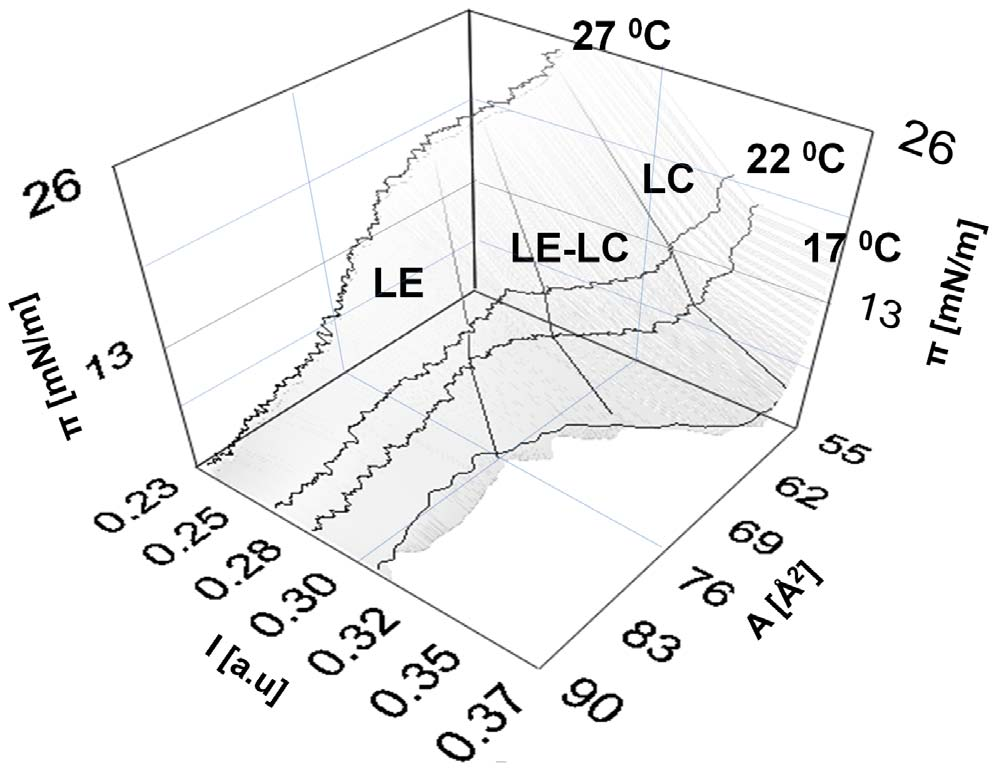
Opto-Mechanical State diagram. The surface can be broadly divided in to three regions as liquid expanded (LE), liquid expanded – liquid condensed (LE-LC) and liquid condensed (LC) region. Slopes along the *A* axis represent compressibility and area fluctuations, while slopes along *I* characterize opto-mechanical susceptibility and fluctuations in Intensity, respectively. The transition region (LE-LC) moves to higher pressure, lower area and lower intensity with increasing temperature. The curves represent real data acquired using DPPC-NBD.

The correlation is particularly impressive close to their maxima. This is where the lipid monolayer is near or in its LE-LC coexistence region. The similarity between the two curves also suggests that during the transition, area and intensity changes are approximately proportional *δI/I ∼ δA/A*. This proportionality is in fact shown to be conserved for transitions at different temperatures (fig. 3). From a thermodynamic perspective this implies that the intensity is an extensive observable of the lipid-dye system. In Fig. 4a the coupling curves are plotted for a different lipid system (DMPC) at a different temperature and the correlation is found to be conserved showing that the correlation is a property of the thermodynamic state and *not* the nature of molecules. We should mention, that some of the curves, in particular those far from room temperature, exhibited a slight mismatch between the peaks in compressibility and opto-mechanical susceptibility. The source of this mismatch is temperature gradients within the monolayer. To test this, we measured the coupling at different locations along a defined temperature gradient. In fig. 4b, we see that the mismatch clearly follows the temperature perturbation. The temperature difference of ΔT = 0.8°C corresponds to a pressure difference of 1.5 mN/m (from 18 mN/m to 19.5 mN/m). This is in perfect agreement with the isotherms (fig. 4b, inset). *This data demonstrates that fluorescence intensity can report locally varying thermodynamic properties in lipid membranes.* Indeed, the interrelationships of area, intensity, pressure and temperature can be presented in a 3D state diagram (fig. 5).

### 2. Non-equilibrium Opto-mechanical Coupling

**Figure 6:**
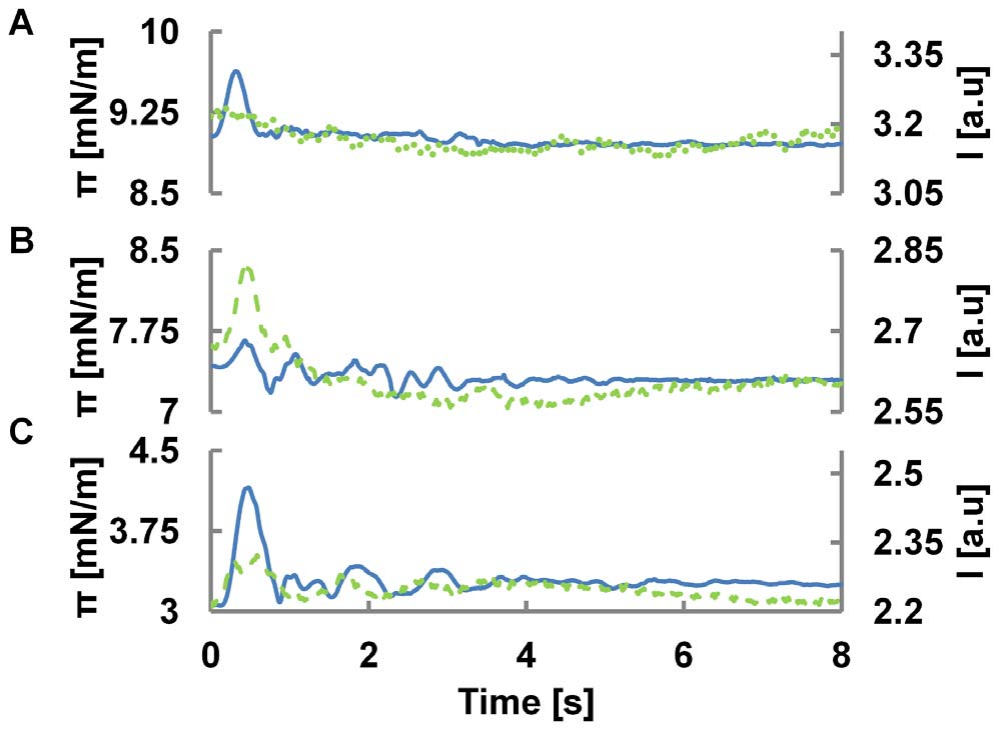
Optical measurement of a Pressure Pulse . The state of the initial equilibrium is marked on the pressure intensity isotherm in top left. Time profile of intensity (green-dashed) v/s pressure (blue-solid) pulses is plotted in A) LC, B) LE-LC and C) LE region. In the LE region intensity moderately couples to a propagating pressure pulse. Intensity is most sensitive to small pressure variations in LE-LC region. This non-equilibrium behavior is qualitatively similar to the quasi-static coupling. The opto-mechanical coupling under non-equilibrium conditions suddenly disappears in the LC regions where the intensity hardly responds to much stronger pressure variations. In (B) the pulse arrives at Δt = 0.44s at a distance of 12cm giving a velocity of propagation c = 0.27 m/s. Experiments were performed in a DPPC/NBD-PE (1%) system at 21°C.

**Figure 7:**
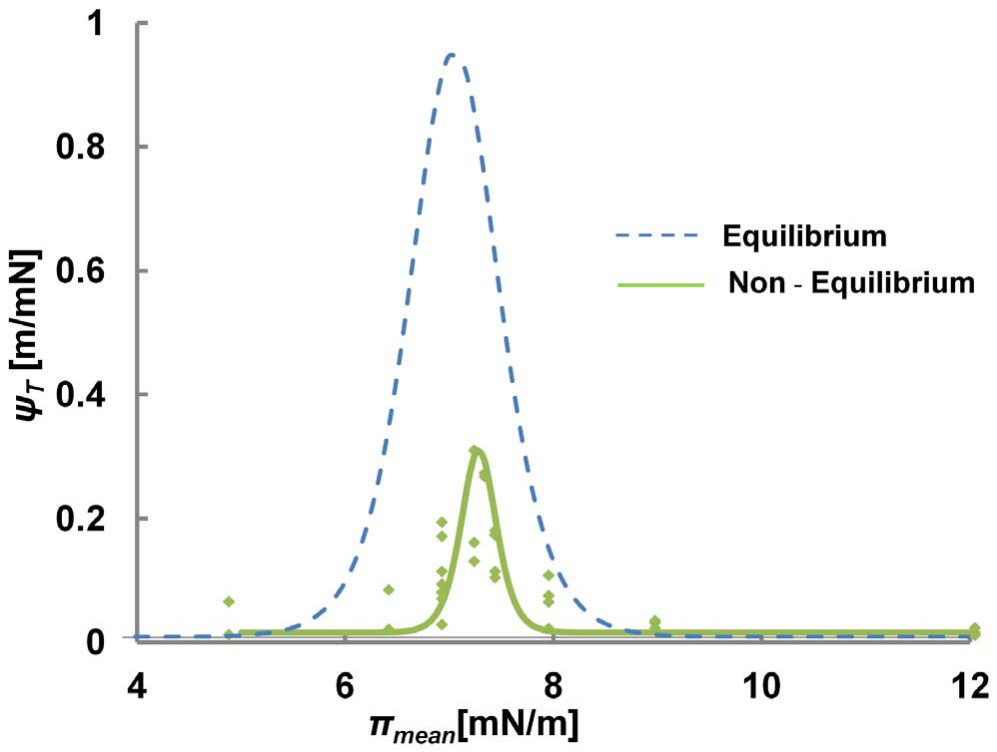
Opto-Mechanical Coupling for Non Equilibrium Processes (Propagating Pulses). Non-equilibrium opto-mechanical susceptibility (green) as obtained from differential changes in pressure and intensity during individual pulses. The corresponding opto-mechanical susceptibility under equilibrium conditions is plotted in blue. Peak in equilibrium as well as non equilibrium conditions appear around 7 mN/m. Both fits were obtained assuming a sigmoidal response of intensity with respect to pressure in the transition region. The fit quality therefore gets worse away from the transition as the priority was to resolve the peak.

In the context of nerve pulse conduction, many fluorescent dyes have been used and developed to follow the propagation of pulses along the axon [23], [24]. Usually these dyes are described as voltage sensitive, changing their fluorescent intensity upon changes in membrane potential [25], [26]. A thermodynamic view on nerve conduction, however, describes the propagation based on sound in two dimensions [4], [27], which implies the propagation of a density oscillation, experimentally confirmed by Tasaki and others [28], [29]. Furthermore, we have recently proposed [1] that propagating density or pressure pulses may be a far more common and fundamental mechanism of communication between proteins, cells and even larger biological entities (e.g. organ or brain areal), *which is not at all limited to nerve pulse propagation*. We demonstrated that density pulses propagating over macroscopic distances could be excited in lipid monolayer [1]. Their velocity follows the state (∼ compressibility) of the membrane and is typically in the range between 0.1–1 m/s, but can also reach the ∼100 m/s range [2]. Furthermore, we have shown that the dielectrical properties of the interface lead to a propagating voltage pulse coupled to the reversible density oscillations of the film [3]. Indeed, thermodynamically, any observable of the interface should undergo similar changes, i.e. a pH pulse, a temperature pulse and an optical pulse is expected as well. As the optical pulse is in particular interesting for its practical implications, we performed non-equilibrium measurements on the opto-mechanical coupling of the monolayer using NBD-PE fluorescent probes. In fig. 6, the correlation between pressure (π(*t*)) and intensity (*I*(*t*)) pulse, simultaneously recorded, is shown for the three fundamentally different states of the system. In the LC phase, the coupling between intensity and pressure is the weakest and only minute changes in intensity can be detected during the propagating pressure pulse. A similar picture appears in the LE phase, where strong pressure pulses couple to intensity pulses of moderate strength. The picture changes completely, when the system is excited within or nearby the transition region, where coupled intensity and pressure pulses are clearly resolved. The opto-mechanical coupling is quantitatively summarized in fig. 7 for both, the equilibrium and non-equilibrium case. Although qualitatively very similar, these curves demonstrate that the extrapolation from equilibrium to non-equilibrium coupling cannot be tacitly assumed, but has to be carefully evaluated. However once established, Fig. 7 allows deriving pressure and compressibility changes from intensity measurements giving clues on the thermodynamic state of the system. For example at a given temperature the non-equilibrium opto-mechanical susceptibility 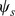 allows to estimate the pressure variations in the membrane from intensity measurements 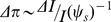. Further, changes in the ratio *ΔI/I, with say* changing pH or temperature, indicate the same quantitative change in *ΔA/A.* Therefore a greater *ΔI/I* would in general imply a softer system. In general, after proper characterization intensity variations can be substituted for hard to measure areal changes. Taking, for example, *ψ_s_* at the maximum value of 0.35 m/mN in Fig. 7, leads to a compressibility of 0.008 m/mN and by applying equ. 4 of reference 1, predicts finally a velocity of 0.32 m/s from optical data (fig. S1). Direct measurements of the propagation velocity (see fig. 6) from the intensity pulse, corresponding to the state of maximum susceptibility, gives a value 0.27 m/s. The agreement between the predicted and the observed velocity underlines that the observed intensity variations are a consequence of changes in thermodynamic state. (Calculations S1).

**Figure 8:**
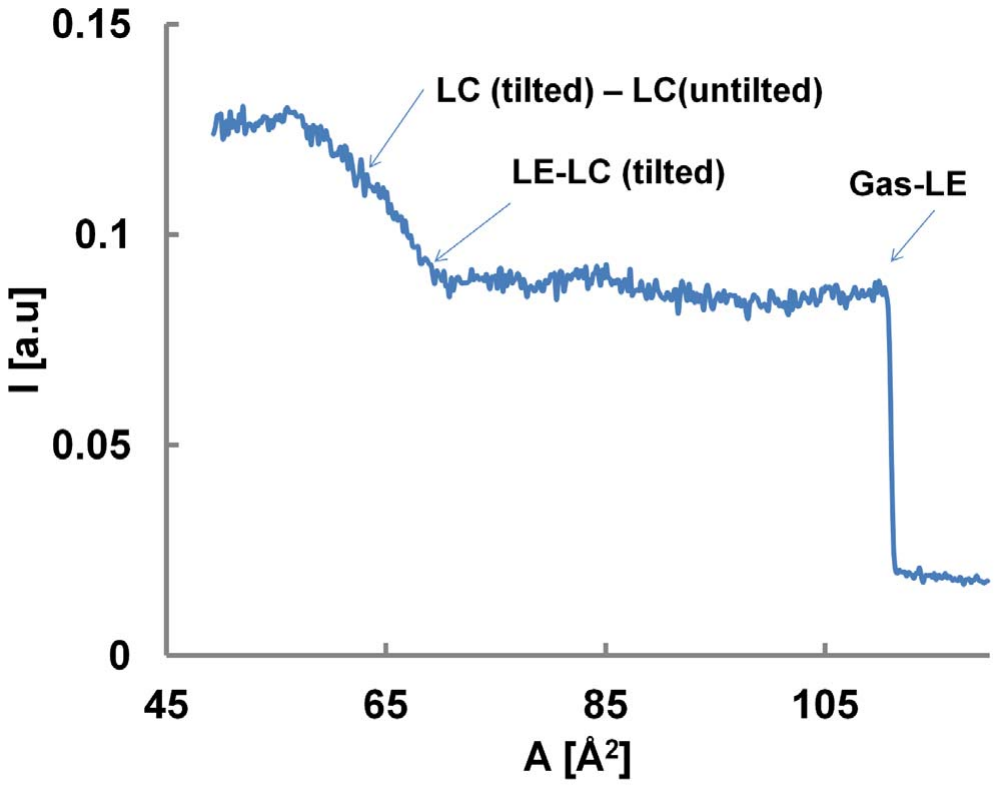
On the Microscopic origin of the Opto-mechanical coupling. Opto-mechanical coupling showing different transitions in a DPPC/NBD system at 22 C. The opto-mechanical isotherm is remarkably similar to a surface potential isotherm for DPPC monolayer. [32] Specifically, the abrupt increase in intensity and the dipole moment during gas-LE transition are precisely aligned and support the dipole based explanation for the microscopic origin of the coupling.

Microscopically the opto-mechanical coupling can be understood by the state dependent electric dipole orientation of the lipid molecules.[30]–[33] The orientation of the absorption dipoles of the dye molecule with respect to the optical field is intrinsically coupled to the orientation of the conjugated lipid molecules. [34] This is impressively demonstrated by comparing the emission intensity and surface potential of the monolayer as a function of area per molecule (fig. 8) (33). The abrupt jump in intensity during gas-LE transition closely corresponds to the jump in surface potential, clearly supporting the dipole based explanation. In the end we should mention that on considering intensity as an observable some aberrations were observed especially at high dye concentration (>0.1%) and small area/molecule (<60 Å^2^) (fig. S2 and S3). These aberrations, namely an unexpected decrease in intensity, are most probably due to self-quenching effects [35]. The general nature of these results was further illustrated by the opto-mechancal isotherms obtained for two other dyes. (fig. S4).

### Conclusion

**Figure 9:**
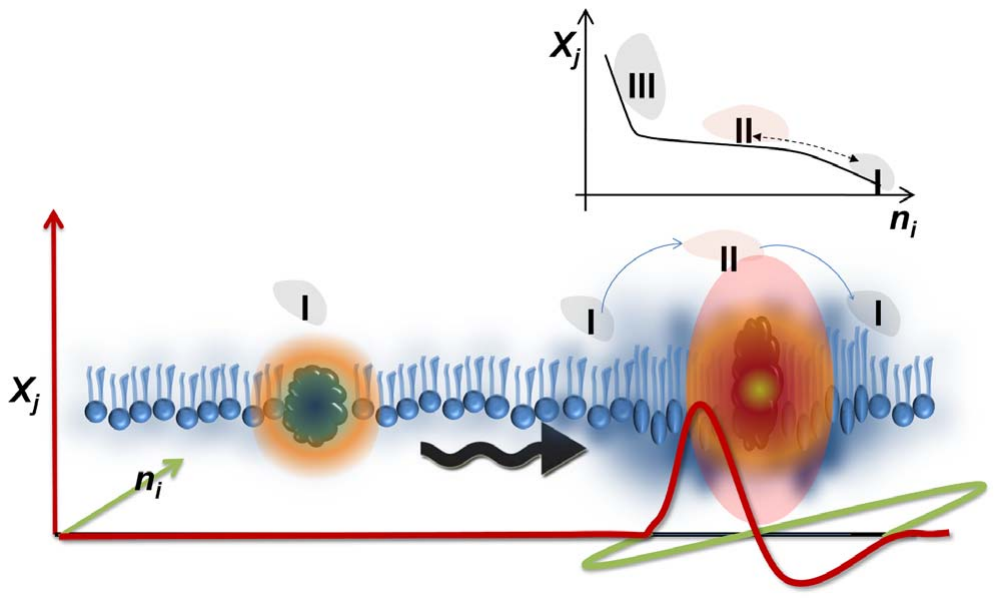
Approximate visualization of Pulse Propagation at an Interface. As a visual aid for beginners, the figure introduces the thermodynamic approach for a propagating pulse at the interface. A perturbation at the interface alters the state of local hydrated environment. The disturbance can propagate and is conserved over long distances influencing the properties of a single molecule (e.g the emission properties of a flourophore, kinetics of an enzyme) located at a remote location. All changes induced are reversible leading to local oscillations in the mean states the wave passes by. In a nonlinear system, the thermodynamic properties can change with a propagating density pulse (I – II). The accompanied state change implies changes in local fluctuations and consequently kinetic processes. Here *X_j_* can be pressure, temperature, electric field etc and *n_i_* can be area, charge, intensity, [H^+^] etc.

Fig. 9 attempts to illustrate the impact of a pulse propagating along an interface. The collective variations in lateral density lead to changes in the emission properties of the incorporated dye. These changes are characterized phenomenologically without stressing molecular models. Thermodynamically, the pulse propagates a change in the local state, expressed by the respective *non-equilibrium* state diagram. The slope of the state diagram in turn determines the fluctuations of the interface which originate from the curvature of the entropy potential of the interface [37], [38]. Although shown here for fluorescently labeled molecules, the dynamic coupling between state and function must exist for other special classes of molecules as well, such as enzymes. In fact the state of the interface has been shown to control the kinetic behavior of membrane bound enzymes [6], [8], [37]–[39],Therefore propagating pulses are predicted to collectively modulate the function of membrane bound enzymes as well. We hypothesize this as a new foundation for cellular signaling: the (catalytic) activity or conformational fluctuations of one protein may initiate a local disturbance, which propagates along the interface. A second protein, exposed to the propagating pulse will respond to the corresponding state changes. Near extreme nonlinearities in the state diagrams (e.g. susceptibility maxima), alterations in protein activity are expected to be significant or even of “*on/off*” kind. Any communication of this form will not require any energy since pulse propagation is adiabatic and enzymes work reversibly.

Our results demonstrate that fluorescence intensity measurements aptly detect abrupt state changes. When near a maxima in susceptibility (as for instance the LE-LC transition), i.e. in the non-linear regime of the interface, the opto-mechanical coupling is very strong hopefully enabling us to study similar state changes in biological interfaces as well. Combined with single molecule measurements, such studies will allow us to demonstrate that non-localized, propagating pulses are capable of controlling the activity of localized single molecules (e.g. enzymes) by transiently changing the thermodynamic state of the environment and provide evidence for a fundamentally new mechanism for the communication between biological entities in general.

## Supporting Information

Figure S1 **Opto-mechanical coupling coefficient.** Intensity and surface pressure as a function of area per molecules for a DPPC-NBD monolayer during a quasi-static compression. The plateau in the pressure curve, which represents the LE-LC coexistence region, is correlated with an abrupt but steady rise in intensity indicating intensity is most sensitive to small pressure variations in this region of the state diagram.The intensity plot also represents a transition around 57 Å^2^ which is not easily identified in pressure data and is most likely the well documented tilted – untilted transition [40]. The drop in intensity towards the small surface areas most likely results from self quenching of the dyes as discussed later. (b) Coupling Coefficient *K(π,T) = −(*Δ*I/I)/(* Δ*A/A)* for DPPC/NBD-PE system (1%) at T = 21°C. The peak value of 30 corresponds to the transition pressure of 7.3 mN/m indicating strongest coupling between area and intensity at the transition. The peak value of 30 corresponds to the transition pressure of = 7.3 mN/m. The coupling coefficient, *K*, is a function of pressure and temperature of the interface. For the non equilibrium calculations, *K* obtained under isothermal conditions was used to obtain *κ_s_* from *ψ_s_* under the quasi-static approximation.
(TIF)

Figure S2 **Intensity-Area isotherms.** Intensity plotted as a function of area per molecule for isotherms of DPPC-NBD monolayer. That the slopes during transition are conserved (fig. 3b) is elaborated here by the parallel nature of the curves. This graph is essentially the projection of the 3D state diagram of figure 3 on the [I-A] plane. Except in the isotherm at 27°C, where before the transition there is a drop in intensity, the intensity mostly increases monotonically with decreasing area per molecule. Among the presented data, the transition occurs at the least value of area per molecule for 27°C.
(TIF)

Figure S3 **Self-quenching in NBD dyes.** DMPC-NBD intensity-area isotherms for a temperature range of 5 to 14 C. The quenching effects are much more pronounced in DMPC-NBD system as compared to DPPC-NBD system. On top is the plot for monolayers with 0.1% NBD. The bottom plot is for NBD concentration of 1% by mole. For the 10 fold increase in concentration of the dye molecule, intensity doesn’t go up by a proportional amount although the order of magnitude is correct. This is the first evidence that there is a concentration dependent quenching in the dye. The increase in concentration amplifies drop in intensity before transition. In both the figures, the effect becomes more dominant as the transition moves to lower area per molecule (marked with solid arrow). The beginning of the first decrease in intensity (marked with dashed arrow) on the other hand seems to be independent of temperature for the given range. On comparing with DPPC in figure S2 the self quenching effects are much more prominent in DMPC, which has a smaller area per molecule. The self quenching in NBD molecules is rather strong as a pure monolayer consisting only of NBD conjugated lipid molecules has negligible intensity response. (data not shown)
(TIF)

Figure S4 **State dependence of the coupling is conserved for different dyes.** Intensity as a function of lateral pressure for DMPC/BODIPY(left) and DMPC/Texas Red (right) (both had 0.1% dye by moles) at several different temperatures. The arrow marks the direction of increasing temperature. The intensity goes through an abrupt increase as a function of surface pressure, same as in NBD. It is repeatable for transitions at several different temperatures for each dye. After accounting for the dip in intensity in BODIPY at higher pressures, the nature of the response of these dyes is qualitatively similar to NBD overall as well. Although the opto-mechanical coupling is a property of the state of the interface, the absolute magnitude and the sign of the coupling coefficient k depends on the particular lipid-dye system.
(TIF)

Calculations S1 **Non-equilibrium Opto-mechanical coupling.** The note explains the relationship between the mechanical and optical data. Opto-mechanical data can be used to correctly estimate the velocity of propagation, which is also accessible experimentally, showing the self consistency of the approach.
(DOCX)
