# Supplementary figures and images for: Opto-Mechanical Coupling in Interfaces under Static and Propagative Conditions and Its Biological Implications
Source: PLoS One. 2013 Jul 4;8(7):e67524. doi: 10.1371/journal.pone.0067524 (PMC3701664; doi:10.1371/journal.pone.0067524)

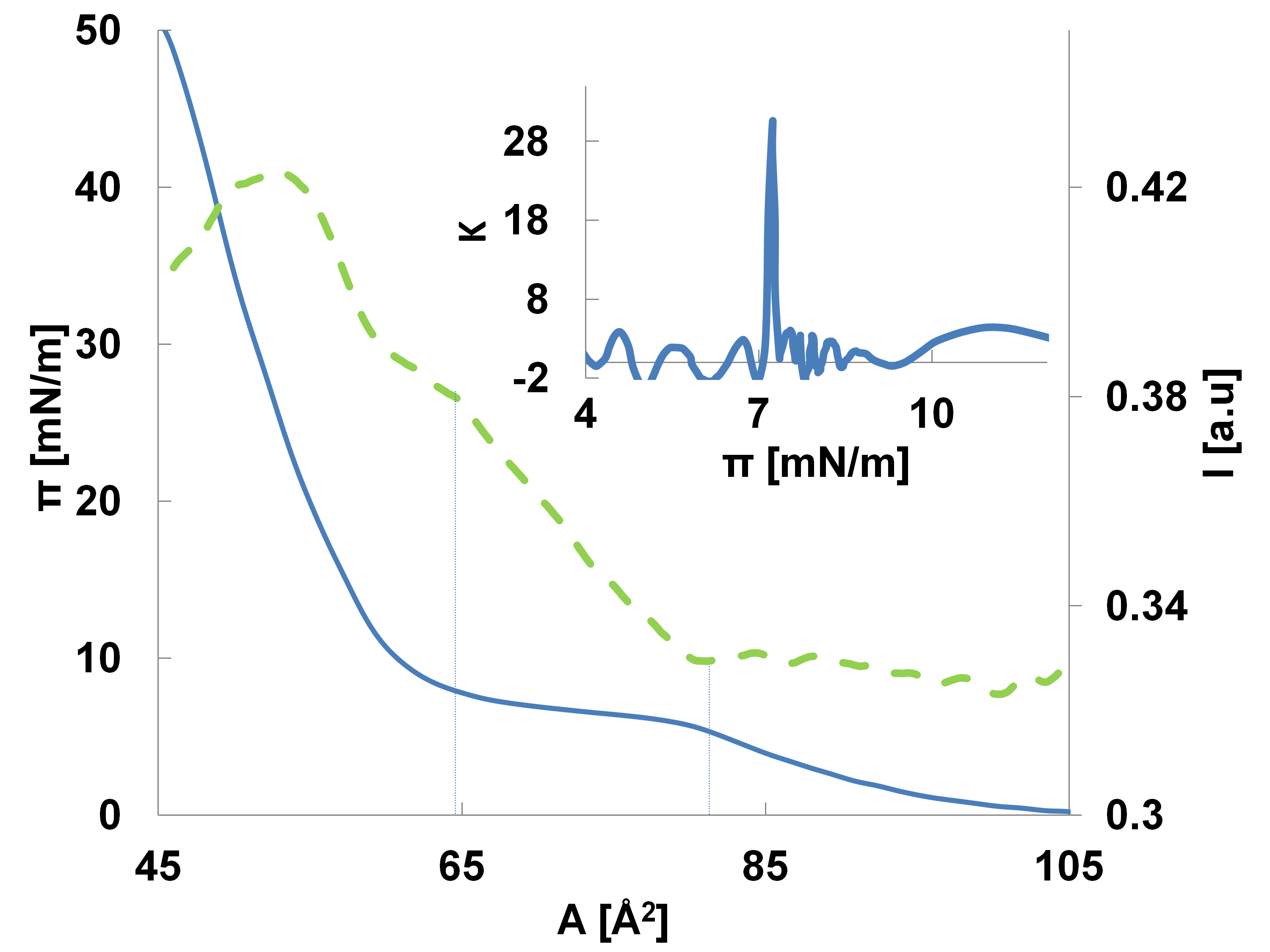

Supplement: Figure S1 — Opto-mechanical coupling coefficient. Intensity and surface pressure as a function of area per molecules for a DPPC-NBD monolayer during a quasi-static compression. The plateau in the pressure curve, which represents the LE-LC coexistence region, is correlated with an abrupt but steady rise in intensity indicating intensity is most sensitive to small pressure variations in this region of the state diagram.The intensity plot also represents a transition around 57 Å2 which is not easily identified in pressure data and is most likely the well documented tilted – untilted transition [40]. The drop in intensity towards the small surface areas most likely results from self quenching of the dyes as discussed later. (b) Coupling Coefficient K(π,T) = −(ΔI/I)/( ΔA/A) for DPPC/NBD-PE system (1%) at T = 21°C. The peak value of 30 corresponds to the transition pressure of 7.3 mN/m indicating strongest coupling between area and intensity at the transition. The peak value of 30 corresponds to the transition pressure of = 7.3 mN/m. The coupling coefficient, K, is a function of pressure and temperature of the interface. For the non equilibrium calculations, K obtained under isothermal conditions was used to obtain κs from ψs under the quasi-static approximation. (TIF) [file pone.0067524.s001.tif]

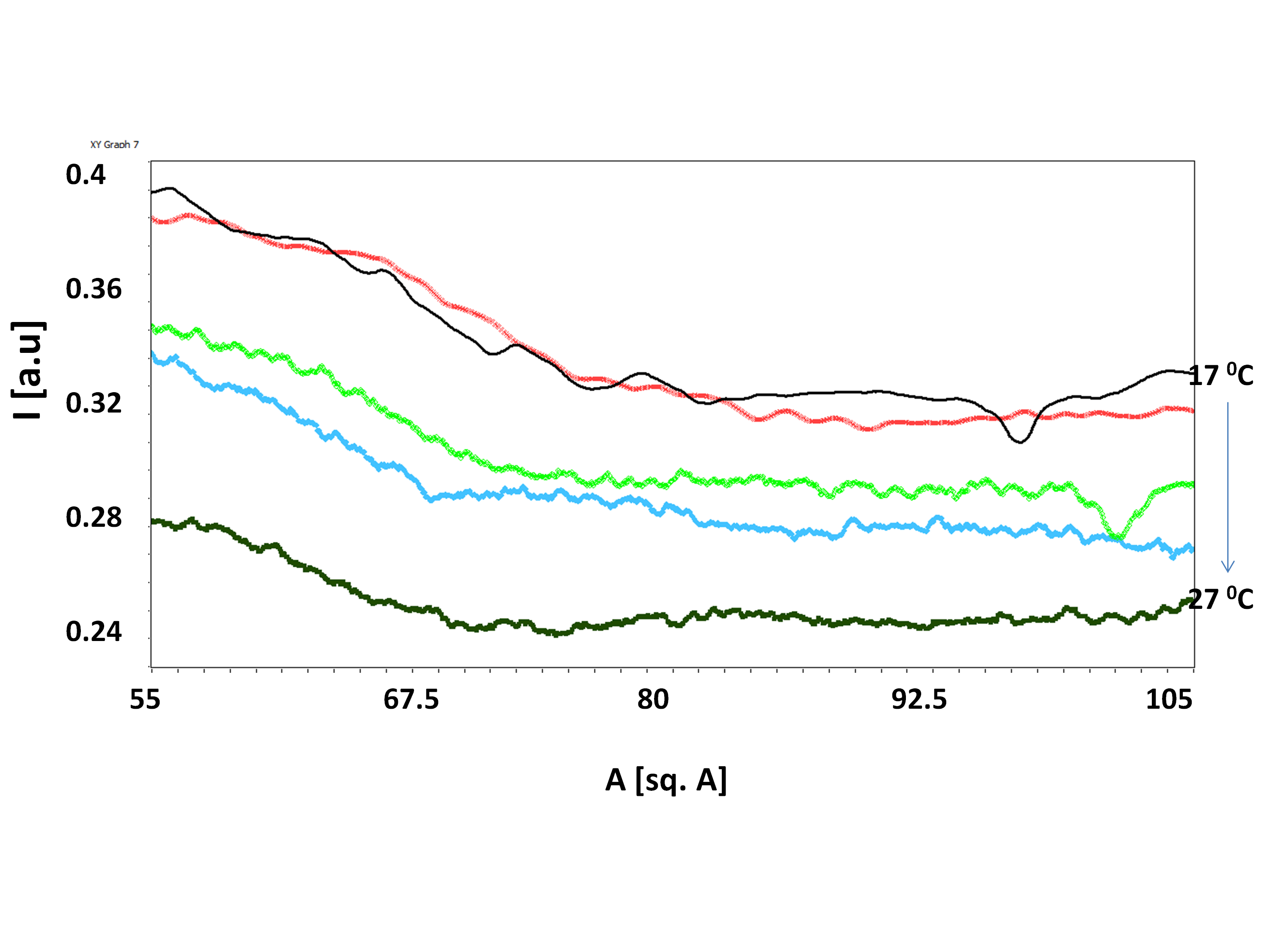

Supplement: Figure S2 — Intensity-Area isotherms. Intensity plotted as a function of area per molecule for isotherms of DPPC-NBD monolayer. That the slopes during transition are conserved (fig. 3b) is elaborated here by the parallel nature of the curves. This graph is essentially the projection of the 3D state diagram of figure 3 on the [I-A] plane. Except in the isotherm at 27°C, where before the transition there is a drop in intensity, the intensity mostly increases monotonically with decreasing area per molecule. Among the presented data, the transition occurs at the least value of area per molecule for 27°C. (TIF) [file pone.0067524.s002.tif]

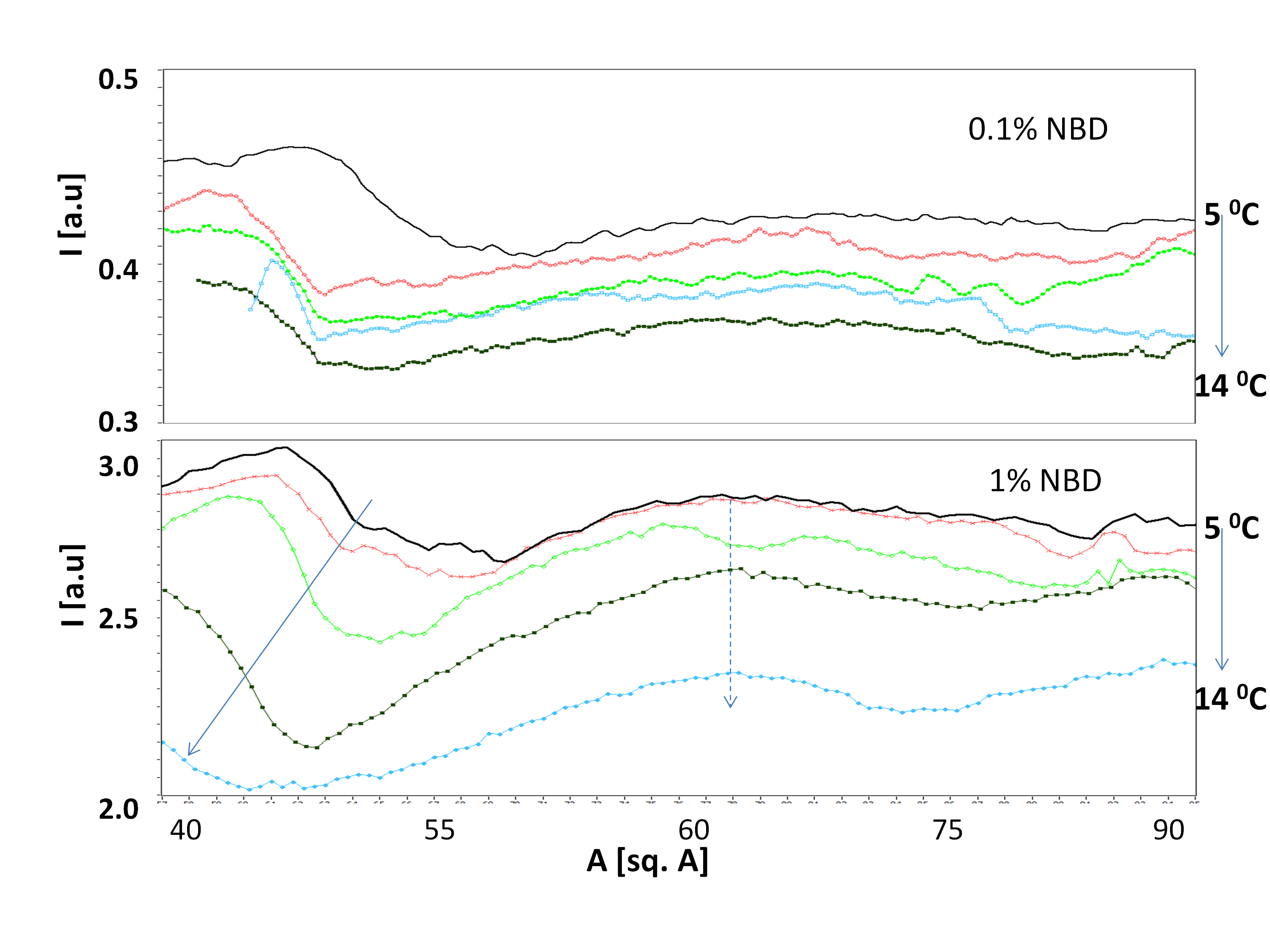

Supplement: Figure S3 — Self-quenching in NBD dyes. DMPC-NBD intensity-area isotherms for a temperature range of 5 to 14 C. The quenching effects are much more pronounced in DMPC-NBD system as compared to DPPC-NBD system. On top is the plot for monolayers with 0.1% NBD. The bottom plot is for NBD concentration of 1% by mole. For the 10 fold increase in concentration of the dye molecule, intensity doesn’t go up by a proportional amount although the order of magnitude is correct. This is the first evidence that there is a concentration dependent quenching in the dye. The increase in concentration amplifies drop in intensity before transition. In both the figures, the effect becomes more dominant as the transition moves to lower area per molecule (marked with solid arrow). The beginning of the first decrease in intensity (marked with dashed arrow) on the other hand seems to be independent of temperature for the given range. On comparing with DPPC in figure S2 the self quenching effects are much more prominent in DMPC, which has a smaller area per molecule. The self quenching in NBD molecules is rather strong as a pure monolayer consisting only of NBD conjugated lipid molecules has negligible intensity response. (data not shown) (TIF) [file pone.0067524.s003.tif]

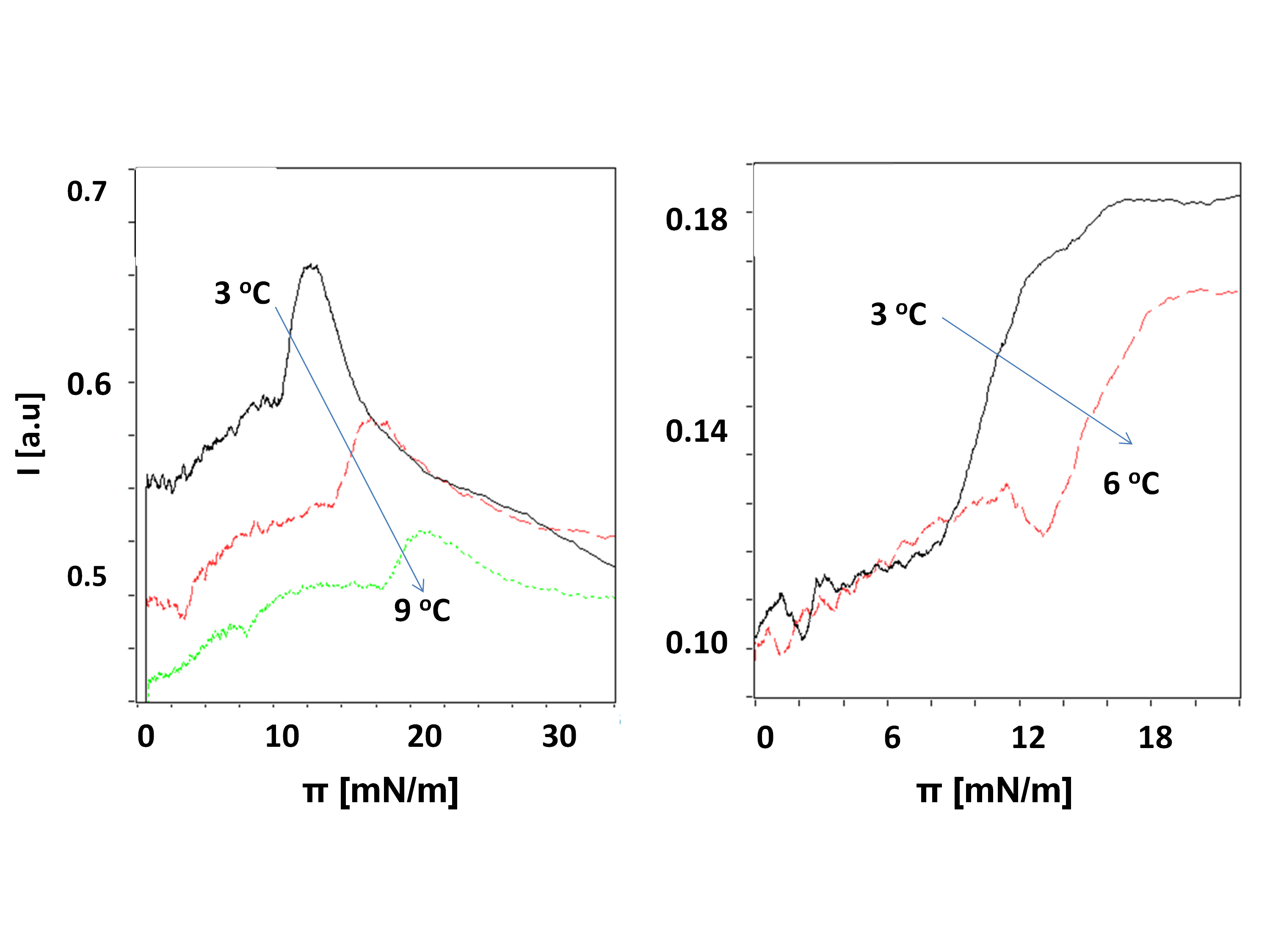

Supplement: Figure S4 — State dependence of the coupling is conserved for different dyes. Intensity as a function of lateral pressure for DMPC/BODIPY(left) and DMPC/Texas Red (right) (both had 0.1% dye by moles) at several different temperatures. The arrow marks the direction of increasing temperature. The intensity goes through an abrupt increase as a function of surface pressure, same as in NBD. It is repeatable for transitions at several different temperatures for each dye. After accounting for the dip in intensity in BODIPY at higher pressures, the nature of the response of these dyes is qualitatively similar to NBD overall as well. Although the opto-mechanical coupling is a property of the state of the interface, the absolute magnitude and the sign of the coupling coefficient k depends on the particular lipid-dye system. (TIF) [file pone.0067524.s004.tif]
