# Supplementary material for: Opto-Mechanical Coupling in Interfaces under Static and Propagative Conditions and Its Biological Implications
Source: PLoS One. 2013 Jul 4;8(7):e67524. doi: 10.1371/journal.pone.0067524 (PMC3701664; doi:10.1371/journal.pone.0067524)
Supplement: Calculations S1 — Non-equilibrium Opto-mechanical coupling. The note explains the relationship between the mechanical and optical data. Opto-mechanical data can be used to correctly estimate the velocity of propagation, which is also accessible experimentally, showing the self consistency of the approach. (DOCX) [file pone.0067524.s005.docx]

**Calculations**

π and I are accessible during a propagating pulse, approximating their variations as differentials, we get;

$\frac{I\Delta\pi}{\Delta I}\approx\left(\frac{Id\pi}{dI}\right)_{S}$ (1)

Subscript S signifies that under ideal conditions the process would be of adiabatic nature. For low frequencies (here ω~1Hz) we further approximate

$\left(\frac{\partial A}{\partial I}\right)_{S}\approx\left(\frac{dA}{dI}\right)_{T}$ (2)

The right hand side of eq.2 is calculated experimentally (Fig. S1). The adiabatic compressibility is defined as

$\kappa_{S}\equiv-\frac{1}{A}\left(\frac{\partial A}{\partial\pi}\right)_{S}$ (3)

Thus we can estimate the dynamic compressibility from the opto-mechanical data by using equation 1,2 and 3

The compressibility can then be substituted in to the expression previously derived for pressure pulse velocity in lipid monolayers [1].

$c=\frac{1}{\cos\left(\frac{\pi}{8}\right)}\sqrt{\frac{1}{\kappa_{S}}\sqrt{\frac{\omega}{\eta_{W}\rho_{W}}}}$ (4)

The left hand side is also accessible from pure intensity measurement. We would like to mention that eq 1-3 should in general be applicable in any system but equation 4 was derived specifically for lipid monolayer. It is therefore highly dependent on the constraints and assumptions involved and should be used with caution after careful deliberation of the corresponding citation [1].
